# Ablation of Ca_V_2.1 Voltage-Gated Ca^2+^ Channels in Mouse Forebrain Generates Multiple Cognitive Impairments

**DOI:** 10.1371/journal.pone.0078598

**Published:** 2013-10-31

**Authors:** Robert Theodor Mallmann, Claudio Elgueta, Faten Sleman, Jan Castonguay, Thomas Wilmes, Arn van den Maagdenberg, Norbert Klugbauer

**Affiliations:** 1 Institut für Experimentelle und Klinische Pharmakologie und Toxikologie, Albert-Ludwigs-Universität, Freiburg, Germany; 2 Fakultät für Biologie, Albert-Ludwigs-Universität, Freiburg, Germany; 3 Institut für Physiologie II, Albert-Ludwigs-Universität, Freiburg, Germany; 4 Departments of Human Genetics and Neurology, Leiden University Medical Centre, Leiden, The Netherlands; School of Medicine and Health Sciences, University of North Dakota, United States of America

## Abstract

Voltage-gated Ca_V_2.1 (P/Q-type) Ca^2+^ channels located at the presynaptic membrane are known to control a multitude of Ca^2+^-dependent cellular processes such as neurotransmitter release and synaptic plasticity. Our knowledge about their contributions to complex cognitive functions, however, is restricted by the limited adequacy of existing transgenic Ca_V_2.1 mouse models. Global Ca_V_2.1 knock-out mice lacking the α1 subunit *Cacna1a* gene product exhibit early postnatal lethality which makes them unsuitable to analyse the relevance of Ca_V_2.1 Ca^2+^ channels for complex behaviour in adult mice. Consequently we established a forebrain specific Ca_V_2.1 knock-out model by crossing mice with a floxed Cacna1a gene with mice expressing Cre-recombinase under the control of the *NEX* promoter. This novel mouse model enabled us to investigate the contribution of Ca_V_2.1 to complex cognitive functions, particularly learning and memory. Electrophysiological analysis allowed us to test the specificity of our conditional knock-out model and revealed an impaired synaptic transmission at hippocampal glutamatergic synapses. At the behavioural level, the forebrain-specific Ca_V_2.1 knock-out resulted in deficits in spatial learning and reference memory, reduced recognition memory, increased exploratory behaviour and a strong attenuation of circadian rhythmicity. In summary, we present a novel conditional Ca_V_2.1 knock-out model that is most suitable for analysing the *in vivo* functions of Ca_V_2.1 in the adult murine forebrain.

## Introduction

Voltage-gated Ca_V_2 Ca^2+^ channels play a key role in depolarization-evoked neurotransmitter release. Both Ca_V_2.1 (P/Q-type) and Ca_V_2.2 (N-type) Ca^2+^ channels initiate the fusion of presynaptic vesicles with the plasma membrane by providing a high transient Ca^2+^ concentration at the active zone of the synapse playing a major role in transmitter release at many excitatory synapses of the central nervous system [1], [2]. Ca_V_2.1 channels are located closer to the vesicles at the active zone and may produce a more efficient and precise transmitter-release as shown in the Calyx of Held and other central synapses [3], [4]. Whereas Ca_V_2.2 channels are preferentially active during early development, the relative contribution of Ca_V_2.1 channels to transmitter release increases with postnatal age [5], [6]. Although Ca_V_2.1 and Ca_V_2.2 channels are regulated by second messengers and complex with the synaptic vesicle release machinery, they differ in their regulation and abilities to integrate multiple signalling inputs [7]. Ca_V_2.1 channels have recently been described also in the context with Alzheimeŕs disease since they form molecular targets for soluble amyloid-ß oligomers that progressively accumulate in the brain of Alzheimeŕs disease patients [810].

The role of Ca_V_2.1 channels in cognitive functions has not been well studied. This is partly due to the fact that global ablation of Ca_V_2.1 channels by *Cacna1a* gene deletion in a gene targeting knock-out strategy produces mutant mice with progressive neurological deficits that result in ataxia and dystonia, and that limits survival 3 to 4 weeks after birth [11]–[13]. To different extent, dystonia, ataxia, premature death, and epilepsy have also been observed and extensively studied in natural *Cacna1a* gene mouse mutants *leaner, tottering, rolling Nagoya* and *rocker* [14], [15]. In humans, *Cacna1a* gene mutations are associated with familial hemiplegic migraine type 1, episodic ataxia type 2 and spinocerebellar ataxia type 6 [16]–[18].

To overcome the limitations of global knock-out Ca_V_2.1 models, we took advantage of mice with a floxed *Cacna1a* allele [19] and crossed them with animals expressing Cre-recombinase under the control of the NEX promoter. In the latter, Cre-activity is most prominent in the neocortex and the hippocampus, and within the dorsal telencephalon Cre-mediated recombination is confined to pyramidal neurons, hilar mossy cells and dentate gyrus granule cells [20]. *Cacna1a* gene deletion based on NEX promotor driven Cre-expression results in viable mice lacking Ca_V_2.1 channels selectively in brain regions that have been shown to be important for learning and memory, but not in regions that are considered to be crucial for motor coordination, such as the cerebellum. This strategy led to the generation of viable, conditional Ca_V_2.1 knock-out (cKO) mice that were subjected to a comprehensive set of well-established behavioural tasks.

## Materials and Methods

### Housing of animals

All animal experiments were performed in compliance with the German animal protection law (TierSchG). Mice were housed and handled in accordance with good animal practice as defined by FELASA (www.felasa.eu/guidelines.php) and the national animal welfare body GV-SOLAS (www.gv-solas.de/index.html). The animal welfare committee of the University of Freiburg as well as local authorities (Regierungspräsidium Freiburg) approved all animal experiments. Animals - groups of 2-6 mice per cage - were housed in a temperature and humidity controlled vivarium with a 12 h light-dark cycle, food and water were available *ad libitum*. To exclude possible influences of complex environmental enrichment on behavior only nest-building material was available to the animals [21]. All behavioral experiments were performed during day time.

### Generation of conditional Ca_V_2.1 knock-out mice

In order to generate a forebrain-specific knock-out mouse model of Ca_V_2.1 (cKO) we crossed mice harbouring a floxed *Cacna1a* allele in which exon 4 was flanked by LoxP sites [19] with mice expressing Cre-recombinase under the control of the *NEX* promoter [20]. We have previously demonstrated the effectiveness of this strategy for generating a spatially-restricted knock-out of Ca_V_1.2 [22].

### Western blot analysis

To investigate the expression of Ca_V_2 channels in mice, we developed new rabbit polyclonal antibodies against murine Ca_V_2.1 and Ca_V_2.2 α1 subunits. Both peptide epitopes were derived from part of the loop between domain 2 and 3: Ca_V_2.1_(885–901)_ QQREHAPPREHAPWDAD and Ca_V_2.2_(997–1013)_ NAVEGDKETRNHQPKEP. The Ca_V_1.2 antibody was first described in reference [23]. The antibodies were used in a concentration ranging from 0.1–1.0 µg/ml. As a control we assessed Na^+^/K^+^-ATPase expression (Abcam, Cambridge, UK). Secondary antibodies were obtained from mouse and rabbit IgG-POX (Biomol, Hamburg, Germany).

Membrane-preparations (each 10 µg) were obtained from neocortex, hippocampus or cerebellum of mouse brain and were separated on a 7% SDS-polyacrylamide gel. The gel was blotted using a “wet-blot-chamber” (Bio-Rad Laboratories, München, Germany) on a nitrocellulose membrane (Carl-Roth, Karlsruhe, Germany). After blocking and washing the membranes were incubated with the primary antibody and after washing the secondary antibodies were applied. The proteins were detected using an imaging system (LAS-3000 mini, Fujifilm-Europe, Düsseldorf, Germany) according to the manufacturer's instructions.

### Immunohistochemistry

Deeply anesthetized mice were perfused through the left ventricle with 0.9% NaCl, followed by 4% paraformaldehyde in 0.1 M phosphate buffer, pH 7.4. Brains were removed and postfixed overnight in the same fixative before vibratome sectioning. Immunohistochemical analysis was performed on horizontal free-floating vibratome sections (50 µm).

All sections were incubated with blocking solution (phosphate buffer containing 1% Triton X-100, 1% bovine serum albumin and 5% normal goat serum) for 1h. Sections were incubated for 3h at room temperature with Ca_V_2.1 Ab (5 µg/ml in blocking solution) and then at 4°C overnight. After washing with phosphate buffer, sections were incubated with the secondary Ab (Cy3-biotinylated goat anti-rabbit IgG, diluted 1:800) with 1% BSA and 0.1% Triton X-100 for 2 h at room temperature. Sections were then washed and stained with 4', 6-Diamidino-2-phenylindole, [DAPI]. After final washes in phosphate buffer slices were mounted with Immu-Mount (Thermo Scientific Shandon). Images were taken with an ApoTome fluorescence microscope (Carl Zeiss, Göttingen, Germany) using AxioVision software.

For morphological identification of electrophysiologically analysed cells, brain slices were fixed in 4% paraformaldehyde for subsequent labelling with Alexa-647 conjugated streptavidin (1:1000). The morphology of the stained neurons was assessed with a LSM 510 laser-scanning microscope (Carl Zeiss, Göttingen, Germany).

### Electrophysiology

CTR (control) and cKO mice aged between 7–38 weeks were decapitated under deep isoflurane anaesthesia. Brains were transferred to an ice-cold solution containing (in mM) 87 NaCl, 25 HCO_3_, 2.5 KCl, 1.25 NaH_2_PO_4_, 10 glucose, 75 sucrose, 0.5 CaCl_2_, and 7 MgCl_2_ (equilibrated with 95% O_2_/5% CO_2_). Transverse hippocampal slices (300 µm) and sagittal cerebellum slices (200 µm) were cut using a vibratome (VT-1200, Leica, Germany) and allowed to recover for 30–60 min at 34°C in artificial cerebro spinal fluid (ACSF) consisting of (in mM) 125 NaCl, 25 NaHCO_3_, 2.5 KCl, 1.25 NaH_2_PO_4_, 25 glucose, 2 CaCl_2_, and 1 MgCl_2_ (equilibrated with 95% O_2_/5% CO_2_), and then stored at 20–23°C. Whole-cell patch-clamp recordings were performed using pipettes pulled from borosilicate glass (Hilgenberg GmbH, Malsfeld, Germany; outer diameter 2 mm, inner diameter 1 mm) with a resistance of 3–7 MΩ when filled with a solution containing (in mM) 110 K-gluconate, 40 KCl, 2 MgCl_2_, 10 HEPES, 0.1 EGTA and 2 Na_2_ATP (pH 7.2). Biocytin (1–2 mg/ml) was added for *post-hoc* visualization and morphological identification of the recorded cells. Signals were recorded using a Multiclamp 700B amplifier (Molecular devices, Sunnyvale, USA). Voltage-clamp signals were filtered at 5 kHz with a four-pole low-pass Bessel filter and digitized at 40 kHz using a Power1401 data acquisition interface (Cambridge electronic design, Cambridge, UK) connected to a personal computer and acquired with custom made software written in Igor pro 6 (F-pulse; U. Fröbe, University of Freiburg, Germany). ACSF was supplemented with 5 µM SR95531 to block GABA_A_ receptor mediated currents. The selective Ca^2+^ channel blockers ω-conotoxin GVIA and ω-agatoxin IVA (Bachem AG, Bubendorf, Switzerland) were bath applied using a recirculation system to reduce the total volume to 5–10 ml. Bovine serum albumin (1 mg/ml) was added to the extracellular solution to prevent absorption of the toxins into the perfusion system. Recordings were made at room temperature (∼24°C). CA1 PCs (pyramidal cells) and Purkinje cells were selected using differential interference contrast video-microscopy. Resting membrane potential determined immediately after breakthrough was –64.7±0.8 and –64.6±0.8 mV for CTR and cKO PCs respectively, and –61.4±2.3 mV for Purkinje cells. Extracellular stimulation was performed with glass pipettes filled with Na^+^-rich HEPES solution (stimulus duration: 0.1 ms; frequency: 0.1 Hz) positioned in the stratum radiatum of CA1 or in the molecular layer of the cerebellar cortex. EPSCs were recorded in the voltage-clamp mode at a holding potential of –70 mV for PCs and at –60 for Purkinje cells. Series resistance was 10–25 MΩ and left uncompensated. Experiments in which series resistance changed more than 20% were discarded. Input-output curves were constructed by changing the intensity of extracellular stimulation in a pseudo random order. The resistance of extracellular pipettes was on average 2.3±0.2 and 2.4±0.5 MΩ for CTR and cKO experiments respectively. When testing the effect of channel blockers and when analysing multiple pulse dynamics, stimulus intensity was set to ∼1.5 times the threshold for eliciting an excitatory postsynaptic current (EPSC) for CTR animals, and maximum stimulation in cKO mice, which was necessary to elicit a reliable synaptic response. In several cases no EPSCs were observed even when using the highest stimulus intensity (10 V) in cKO animals, but these experiments were not included in the analysis. Data was analysed using Igor Pro 6 (Wavemetrics, Lake Oswego, OR, USA). Amplitudes of evoked EPSCs were calculated as the average of 20–50 consecutive trials including failures. Early and late multiple pulse facilitation were quantified as ratios between the synaptic charge transferred during defined stimulation periods (∫5^th^ to 10^th^ EPSC/∫1^st^ to 5^th^ EPSC and ∫90^th^ to 100^th^ EPSC/∫1^st^ to 10^th^ respectively). Values are given as mean ± standard error of the mean (SEM). Statistical significance was assessed with a two-tailed student *t*-test and the significance levels are indicated as *P* values.

### Behavioural studies

Activity and behaviour of mice were observed using an automatic video tracking system for recording and analysis (VideoMot2 system V6.01, TSE, Bad Homburg, Germany). Additionally, video-recording software (HaSoTec, FG3xCAP V5.40, Rostock, Germany) and running wheel software (PhenoMaster V3.6.5, TSE, Bad Homburg, Germany) were used. Behavioural data were analysed by using SigmaPlot 12.0 (Erkrath, Germany) and indicated as mean ± SEM. Statistical data of behavioural studies were analysed using one way ANOVA followed by analysis with Student-Newman-Keuls test. Exploratory and anxiety-like behaviour in the open field and activity at open arms in the elevated plus maze test were evaluated using the nonparametric Kruskal-Wallis one way ANOVA on ranks and Dunn's post hoc test**.** P<0.05 was considered as significant.

Control mice (CTR) consisted either of Cacna1a^*flox/flox*^; NEX^*wt/wt*^, Cacna1a^*wt/wt*^; NEX^*wt/cre*^ or Cacna1a^*wt/wt*^; NEX^*wt/wt*^ mice.

One cohort of mice was used to perform the open field and object recognition test, another cohort was used for the elevated plus and Morris water maze. The running wheel and visual tracking drum experiments were performed with both cohorts. Only male mice were used for behavioural studies

### Visual tracking drum

The visual tracking drum consisted of a motorized drum (diameter: 23.5 cm, height: 42 cm), which rotated counter-clockwise with a speed of 2 rev/min. The inner side of the drum was covered with vertical black and white stripes each with 0.4 cm in width. To assess visual performance of the animals, mice were placed on a stationary platform (11 cm in diameter, 19 cm above the bottom of the drum) in the centre of the drum. After a 5 min adaptation-phase on the platform, behaviour of the animals was recorded for 2 min and scored for head tracking movements by at least two independent persons [24], [25].

### Running wheel

Running wheel experiments were performed with animals aged between 105–115 days. Cages with one animal per cage were equipped with running wheels with a regular positioning of bars. Mice were allowed for voluntary wheel running during 14 days. During this time we measured the cumulative running time, average running speed (revolutions/min), distance travelled and running time per 15 min.

### Open field

The open field consisted of a square of 50×50 cm surrounded by a 35 cm wall, illuminated with 65 to 75 Lux. Male mice (p51 to p55) were placed in a centre-square of the peripheral area, with the face orientated to the wall. Behaviour of the animals in the open field was recorded for 20 min. Evaluation of data set included time spent in the central area of the field (10×10 cm squares, 9 central, 16 peripheral), covered distance and average speed in central and peripheral squares.

### Elevated plus maze

The elevated plus maze consisted of two open and two closed arms each of 30×5 cm. Closed arms were surrounded by a 15 cm high wall. All arms emerged from a central platform which was elevated 45 cm above the floor. The maze was made of non-reflecting light grey PVC. Male mice (p70 to p73) were placed on the central platform, facing one of the open arms. The entry and duration of the mice in each arm was continuously assessed during 5 min. Animals that fell when running to the edge of an open arm during the first minute of a session were rapidly placed back onto the open arm and the session was restarted. Animals that crashed more than two times were excluded from the experiment.

### Object recognition task

The object recognition task was performed with male mice aged between 52 to 56 days. The open field box (30×30 cm) was made from non-reflecting grey PVC and was illuminated with 65–75 Lux. Two orbs and a cone were used as objects. Mice were first placed in the empty arena and allowed to freely explore the arena. Then, the animals were submitted to a 10 min acquisition trial in which they were placed in the open field arena in the presence of the two orbs located 8 cm from the left and right wall. After 7 min, the animals were placed again for 5 min in the same arena in which one of the orbs was replaced by a cone. The behaviour of the mice was recorded and evaluated with respect to interaction time and covered distance. Animals were scored as interacting with the objects, when their noses were in contact with the object, or pointing to the object within a defined distance (1.5 cm). Standing, sitting or leaning at/on the objects was not scored as object interaction. The object recognition index (OR_index_) was defined as OR_index_ =  [T_cone_/(T_orb_+T_cone_)]×100; T = interaction time with the object.

### Morris water maze

A circular tank (120 cm diameter) filled with opaque water of 20 to 22°C was positioned in a room with external cues visible to the swimming animal. Male mice (p71 to p74) were repeatedly placed into the pool to locate a submerged escape platform in an initial training phase. Spatial learning was assessed during 5 days across repeated trials, with 4 trials per day. Starting positions varied from trial to trial according to a fixed scheme. After reaching the platform animals were allowed to spend 30 sec on the platform. Animals that were not able to find the platform within 1 min were rescued and placed on the platform for 30 sec.

Reference memory was determined by preference of the platform area when the platform was removed at day 6 of the experiment. Time for platform search was limited to 1 min, after this period animals were rescued from the water. Animals which only `floated (i.e. average swimming speed during the retention trial was below 16 cm/s) for a longer time were excluded from the experiment to guarantee proper evaluation of collected data.

A cued learning task was performed to test the ability of animals to swim straight and target-orientated to a visual stimulus. Cued learning was assessed during 5 days across repeated trials, with 4 trials per day. The submerged platform was cued with a dark-grey object (cone) that extends above the water surface. Starting and platform position varied from trial to trial according to a fixed scheme. After reaching the platform, animals were allowed to spend 30 sec on the platform. Mice that were not able to find/enter the platform within 1 min were rescued and placed on the platform for 30 sec.

## Results

### Basic characterization of conditional Ca_V_2.1 knock-out mice

**Figure 1:**
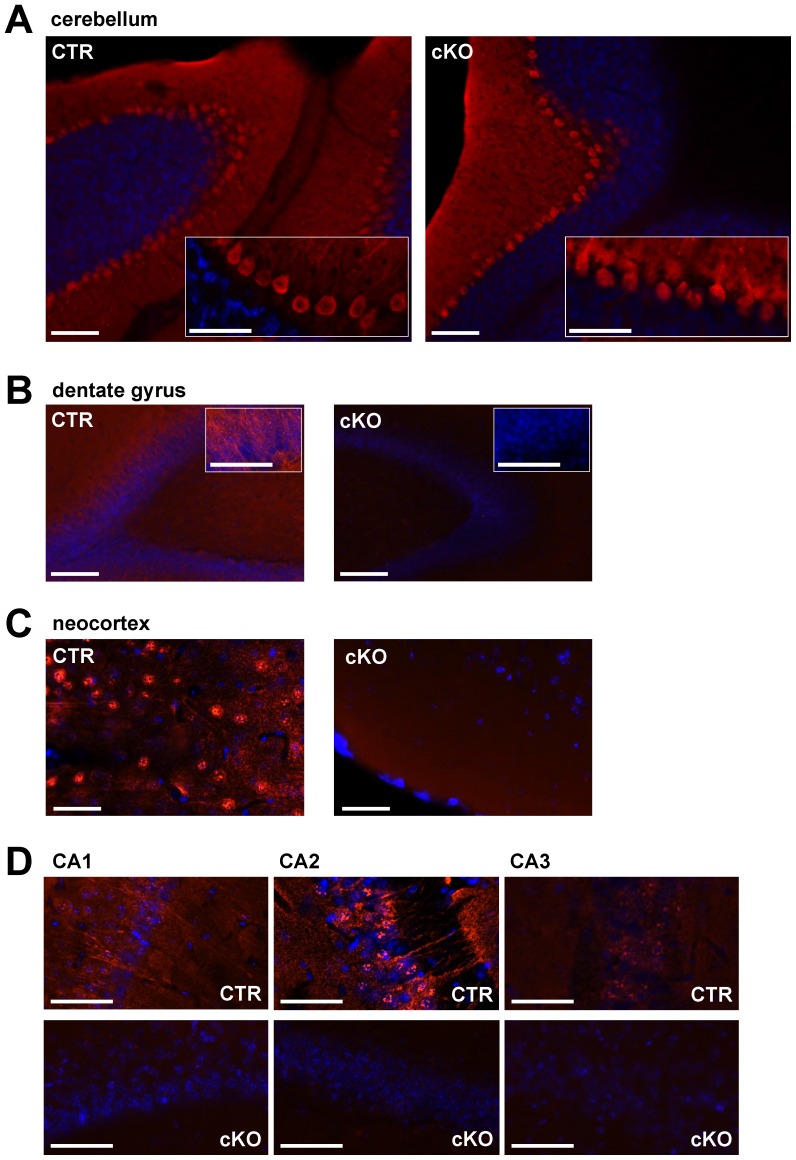
Immunohistochemical comparison of the expression pattern of Ca_V_2.1 in the forebrain and cerebellum of CTR and cKO mice shows a selective knock-out in the hippocampus and neocortex of cKO mice. (A) Ca_V_2.1 immunofluorescence staining in cerebellum demonstrating a high expression of Ca_V_2.1 in Purkinje cell layers of both CTR and cKO mice indicating that the NEX/Cre-mediated knock out strategy did not affect Ca_V_2.1 expression in cerebellum. (B) to (D) Ca_V_2.1 immunofluorescence staining of horizontal sections of the dentate gyrus, neocortex and hippocampal CA1 to CA3 regions demonstrates expression of Ca_V_2.1 throughout CA1, CA2, CA3, dentate gyrus and neocortex in CTR and a hardly detectable expression of Ca_V_2.1 in cKO mice. Ca_V_2.1 expression is shown in red, DAPI in blue. Scale bars: (A) 100 µm (inset 50 µm); (B) 100 µm (inset 50 µm); (C) 50 µm; (D) 50 µm.

The specificity of the NEX/Cre-mediated *Cacna1a* gene deletion was assessed by immunohistochemistry (Fig. 1) and by Western blot analysis (Fig. S1A). The immunohistochemical Ca_V_2.1 α1-subunit staining clearly demonstrated that the NEX/Cre-mediated knock-out strategy successfully worked leading to a hardly detectable Ca_V_2.1 expression in the neocortex, dentate gyrus and hippocampal regions CA1 to CA3 in cKO mice. As expected expression of the Ca_V_2.1 α1 subunit was not affected in the cerebellum (Fig. 1, Fig. S1). Already at an age of p50, we observed a strong reduction of Ca_V_2.1 in the neocortex and hippocampus of cKO mice, and to a minor extent also of heterozygous mice (HET). *Cacna1a* gene deletion was robust in cKO mice and detectable also in one-year-old animals, whereas Ca_V_2.1 expression in cerebellum of cKO mice was not altered. Ca_V_2.1 expression was also not altered in control (CTR) mice. To check for potential compensatory mechanisms by other Ca_V_ channels we evaluated expression of the Ca_V_2.2 α1-subunit (the closest Ca_V_2 member) and of the Ca_V_1.2 α1-subunit (an L-type channel) (Fig. S1B). For both Ca_V_ channels we observed a moderate increase in the hippocampus, but not in the neocortex. This complies with previous observations on the global knock-out of Ca_V_2.1, where N- and L-type currents were increased in Purkinje and cerebellar granule cells [11], [12]. Obviously up-regulation of other channel types is a more general observation since our data are also in line with our previous studies on Ca_V_1.2 knock-out mice, where we found a compensatory up-regulation of Ca_V_1.3. However, the increased expression of Ca_V_1.3 could not compensate the loss of Ca_V_1.2 and animals died during embryogenesis [26].

**Figure 2:**
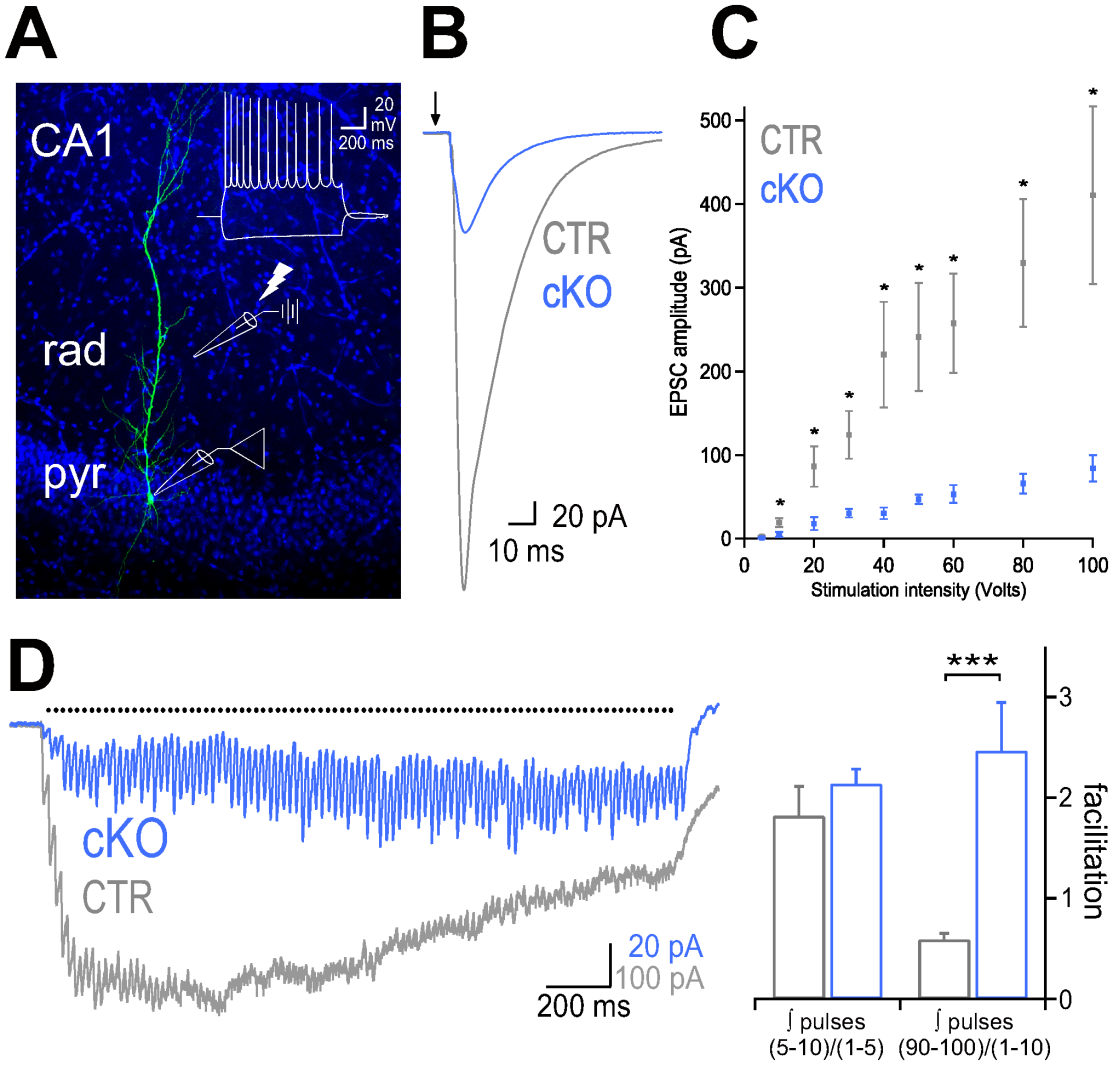
Synaptic transmission at Schaffer collateral-mediated inputs onto CA1 pyramidal cells of cKO mice is impaired. (A) Confocal image stack of an intracellular labeled CA1 pyramidal cell (PC) in a cKO mouse counterstained with DAPI. Inset, superimposed voltage traces of the same PC in response to a 100 pA depolarizing and 50 pA hyperpolarizing current injection (1 s). (B) Representative traces of pyramidal cell EPSCs evoked by extracellular stimulation of Schaffer collaterals (stimulation intensity 100 volts), in CTR (gray) and cKO mice (blue). Arrow marks the time point of stimulation. (C) Summary plot of amplitudes of EPSCs evoked by Schaffer collaterals stimulation at different intensities in CTR (n = 7) and cKO (n = 10) PCs. Means + SEM are shown. (D) Left, average of EPSCs evoked by a train of 100 stimulation pulses at 50 Hz (Black dots indicate the time points of extracellular stimulation, stimulation artifacts have been removed for clarity). Right, synaptic charge transferred during trains of Schaffer collateral-mediated EPSCs is characterized by an early facilitation in both CTR and cKO animals, but strong multiple-pulse facilitation in cKO PCs (n = 9) and multiple-pulse depression in CTR PCs (n = 6) of mice. * *P*≤0.05; ** *P*≤0.01; *** *P*≤0.001 (Two-tailed student *t*-test)

**Figure 3:**
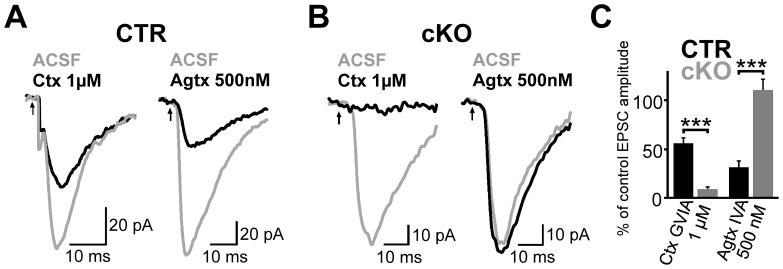
Expression of functional Ca_V_2.1 channels is strongly reduced in the hippocampus of cKO mice. (A and B) Representative EPSCs recorded in CA1 PCs evoked by extracellular stimulation of the stratum radiatum before (gray) and after (black) bath-application of ω-conotoxin GVIA (left) or ω -agatoxin IVA (right). PC recordings were obtained from CTR (A) and cKOs (B). (C) Bar graph summarizes the residual peak amplitude of EPSCs after toxin application for CTR (each 6 experiments) and cKO (5 vs. 6 experiments) mice. Means + SEM are shown. *** *P*≤0.001 (Two-tailed Student’s *t*-test)

To test whether knock-out of Ca_V_2.1 channels may result in alterations of synaptic transmission, we performed whole-cell recordings from CA1 pyramidal cells (PCs) in hippocampal slice preparations of CTR and cKO mice (Fig. 2A) and examined EPSCs evoked by extracellular stimulation of the Schaffer collaterals at different intensities. Input-output curves clearly showed that evoked EPSCs were significantly lower in cKO animals at all stimulation intensities that reliably evoked EPSCs (Fig. 2B and C), suggesting that synaptic transmission at the Schaffer collaterals-CA1 synapse is strongly impaired in cKO animals. To further analyse glutamate release at these synapses, we applied repetitive stimulation at 50 Hz (100 pulses). The synaptic charge transferred during trains of stimulation was by a factor ∼10 smaller in cKOs than CTR mice (Fig. 2D). Facilitation of EPSCs at the onset of the train (∫5^th^ to 10^th^ EPSC/∫1^st^ to 5^th^ EPSC) was similar in CTR and cKO animals but significantly higher at the end of the train (∫90^th^ to 100^th^ EPSC/∫1^st^ to 10^th^) for cKO animals (Fig. 2D). To examine whether the observed reduction in glutamate release from Schaffer collaterals was mediated by a functional loss of P/Q-type Ca^2+^ channels, we bath-applied selective Ca^2+^ channel blockers (Fig. 3A and B) [27]. The N-type Ca^2+^ channel blocker ω-conotoxin GVIA (1 µM) reduced the amplitude of EPSCs in CTR animals to 56.4±5.6%, whereas EPSCs recorded in CA1 PCs of cKO mice were almost entirely blocked by the toxin to a residual signal of 7.6±1.7% (Fig. 3C). Application of the P/Q-type Ca^2+^ channel blocker ω-agatoxin IVA (500 nM) reduced EPSC peak amplitudes in PCs of CTR animals to 31.2±7.3% (5 cells), but had no significant effect on EPSCs recorded in cKO mice (110.5±11.4%; n = 6; Fig. 3C). Thus *NEX*/Cre-mediated loss of Ca_V_2.1 channels resulted in a marked reduction in transmitter release at Schaffer collateral-PC synapses. In contrast, application of ω-agatoxin IVA (500 nM) strongly reduced EPSCs recorded in Purkinje cells after stimulation of parallel fibers to 30.6±6.8% of the control response in cKO animals, further demonstrating the regional specificity of our Ca_V_2.1 KO model.

### Conditional Ca_V_2.1 knock-out mice behaviour

cKO mice did not reveal any behavioural abnormality with respect to motor activity, feeding, nest building-behaviour or any conspicuous feature. We also did not observe spontaneous seizures, stereotypies, increased aggressiveness, poor grooming or any accumulation of injuries. A hanging wire test did not show any difference in grip strength between genotypes (Fig. S2). Body weight of mice at an age of 110 days did not differ between CTR, HET and cKO animals (CTR: 26.7±0.55 g (n = 15), HET: 28.2±0.55 g (n = 12), cKO: 27.6±0.57 g (n = 11)) (Fig. S3). cKO animals at an age of 50 days were marginally lighter than CTR and HET mice (CTR: 21.6±0.33 g (n = 27), HET: 22.9±0.42 g (n = 25), cKO: 20.1±0.37 g (n = 27)).

The visual performance of CTR, HET and cKO mice was analysed using a visual tracking drum. No differences in visual performance indicated by the number of head tracking movements were observed (Fig. S4).

The alterations observed in glutamatergic transmission suggested that cKO mice may have an impaired memory function, changes in their activity or an affected anxiety-related behaviour. This was further analysed in a series of behavioural tests.

### Elevated plus maze

**Figure 4:**
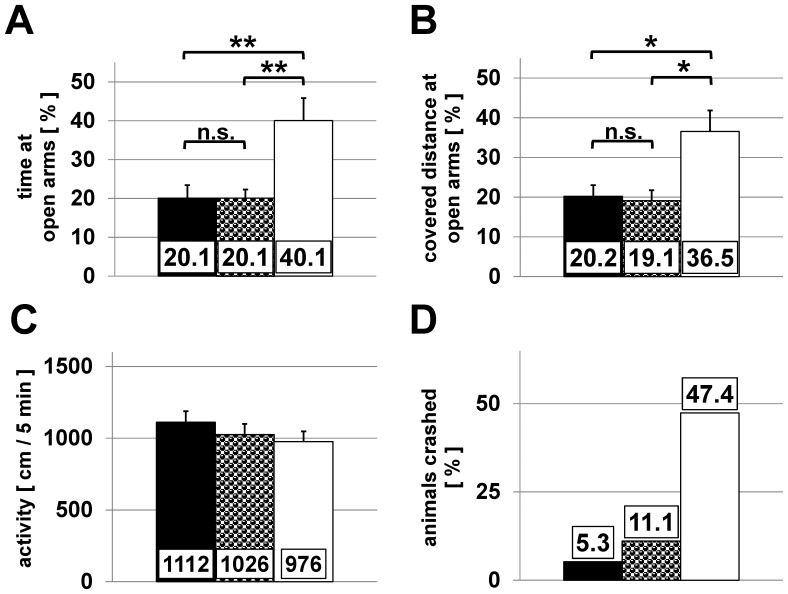
Elevated plus maze. Explorative activity of CTR (black, n = 19), HET (half tone, n = 18) and cKO mice (white, n = 19) was recorded over a time period of 5 min during an elevated plus maze test. Means + SEM are shown for (A) to (C). (A) Percentage of time spent at the open arms of the elevated plus maze. (B) Percentage of the covered distance only at the open arms. (C) Total distance covered during exploration of the elevated plus maze (P = 0.428). (D) Percentage of crashes during the trial. * *P*<0.005; ** *P*<0.003 (Kruskal-Wallis one way ANOVA on ranks & Dunn's post hoc test)

The elevated plus maze is routinely used to study anxiety-related behaviour by measuring the time spending at the open arm area. We found that CTR and HET mice spent about 20.1% (CTR ± 3.4 and HET ± 2.3%) of total time at the open arms whereas cKO mice spent 40.1±5.8% of time at this part of the maze (*P*<0.003) (Fig. 4A). Total covered distance was not significantly different between the genotypes (1112±77.4 cm/5 min (CTR); 1026±73.6 cm/5 min (HET); 976±72.2 cm/5 min (cKO)) (Fig. 4B).

The anti-anxiety behaviour of the cKO mice was also reflected by a comparison of the covered distances. In contrast to cKO mice, CTR and HET mice covered a reduced fraction of the total distance at the open arm (CTR: 20.2±2.8%; HET: 19.1±2.6%; cKO: 36.5±5.3% (*P*<0.005)) (Fig. 4C). The extended habitation of cKO mice at open arms led to a rather high number of falls from the maze (9 of 19 cKO vs. 1 of 19 CTR) (Fig. 4D). Taken together, the elevated plus maze performance of cKO mice may reflect a decreased anxiety-like phenotype and an increased exploratory drive.

### Open field

**Figure 5:**
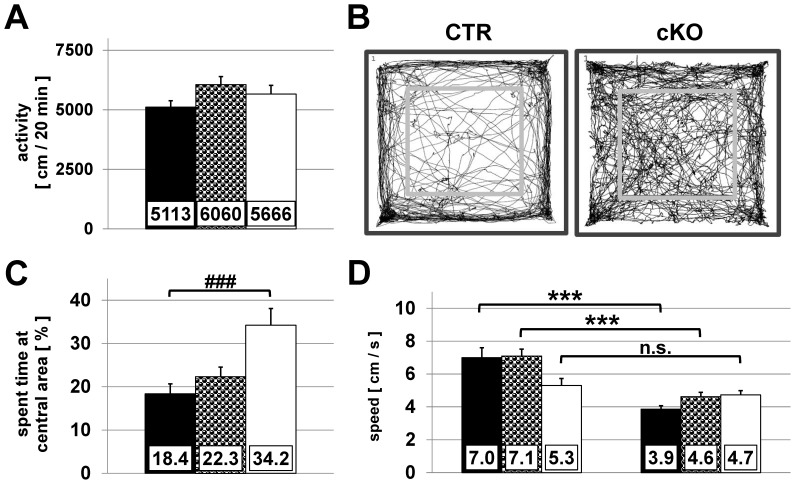
Open field test. Spontaneous activity and exploratory behaviour of CTR (black, n = 17), HET (half tone, n = 16) and cKO mice (white, n = 18) was recorded over a time period of 20 min in the open field arena. Means + SEM are shown. (A) Total distance of movement during the habitation period in the open field (P = 0.138). (B) Illustration of two representative sample tracks from a CTR (left) and cKO (right) animal. (C) Percentage of total time spent in the inner squares of the open field. (D) Average speed of mice during movement within the central (left) and peripheral (right) area. ### *P*<0.01; *** *P*<0.001 (Kruskal-Wallis one way ANOVA on ranks & Dunn's post hoc test)

In the open field arena, activity and anxiety-related behaviour of mice was further analysed. During the 20-min habituation in the open field CTR, HET and cKO mice covered comparable distances ranging from 5113 (±268.9) to 6060 (±336.3) cm suggesting similar level of locomotor activities (Fig. 5A). Typical examples of track records of a CTR and cKO mouse are depicted in Fig. 5B. The time spent in the inner square of the open field is an indicator of anxiety in rodents. As expected, mice avoided entering the centre of the arena and spent on average only 18.4±2.3% (CTR) and 22.3±2.2% (HET), respectively, of the total time in the inner squares (Fig. 5C). In contrast, cKO mice spent 34.2±3.9% of the total time in this area, which closely corresponds to the ratio of the inner over the total area (36%).

Exploratory drive and anxiety of mice is also reflected to some extent by their velocity in the open field (Fig. 5D). CTR and HET mice show a “low” speed during habituation of the outer area, whereas they moved “fast” in the centre area (CTR 3.9±0.2 vs. 7.0±0.6 cm/sec, HET 4.6±0.3 vs. 7.1±0.4 cm/sec; both *P*<0.001). In contrast, cKO mice moved nearly with similar speed in the inner or outer area (5.3±0.4 vs. 4.7±0.28 cm/sec, n.s.). The speed of CTR and cKO mice was significantly different in the inner (7.0 vs. 5.3 cm/sec; *P*<0.05) as well as in the outer (3.9 vs. 4.7 cm/sec; *P*<0.05) area of the arena. In summary, the open field experiment showed that cKOs have similar levels of locomotor activity as CTRs, but have an increased exploratory behaviour.

Next we investigated the consequences of an altered synaptic transmission on learning and memory in cKO mice.

### Object recognition task

**Figure 6:**
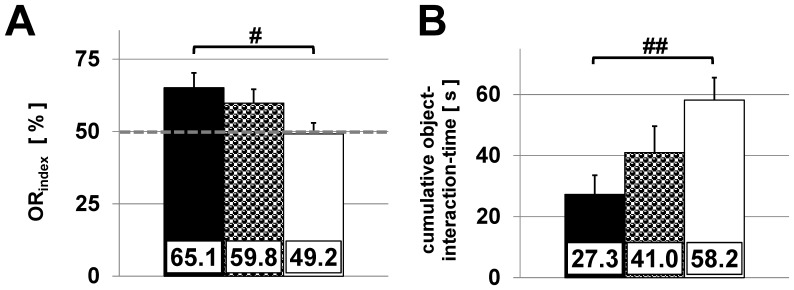
Object recognition task. Evaluation of the non-spatial hippocampal memory of CTR (black, n = 18), HET (half tone, n = 15) and cKO mice (white, n = 18). (A) Object recognition index is defined as follows: The object recognition index (OR_index_) was defined as OR_index_ =  [T_cone_/(T_orb_ + T_cone_)]×100. (T_orb_  =  interaction time with the familiar and T_cone_ interaction time with the new object). Chance level is indicated by the dashed line. Error bars indicate SEM. (B) Total object-interaction time (means + SEM) as an indicator for exploratory drive. # P<0.05; ## P<0.03 (one-way ANOVA & Student-Newman-Keuls post hoc test)

The object recognition task is used to evaluate non-spatial hippocampal memory, i.e. the ability of mice to become acquainted and remember different objects within an open field arena. During acquisition CTR, HET and cKO mice showed no preference for one of the two identical objects and demonstrated no significantly different total interaction times (Fig. S5). The object recognition index derived from the interaction time (Fig. 6A) indicated that CTR mice significantly spent more time with the new object than cKO animals, whose object preference was at chance level (65.1±5.2% (CTR), 59.8±4.8% (HET), 49.2±3.8% (cKO), *P*<0.05).

The total interaction time can be used as an indicator for the exploratory drive of all genotypes and demonstrated that CTR mice spent 27.3±6.3 sec with nosing and exploring of the objects, whereas cKO mice spent 58.2±7.4 sec on average (Fig. 6B). This indicates that cKO mice are not able to differentiate between the objects, but demonstrate a higher exploratory drive than CTR mice.

### Morris water maze

**Figure 7:**
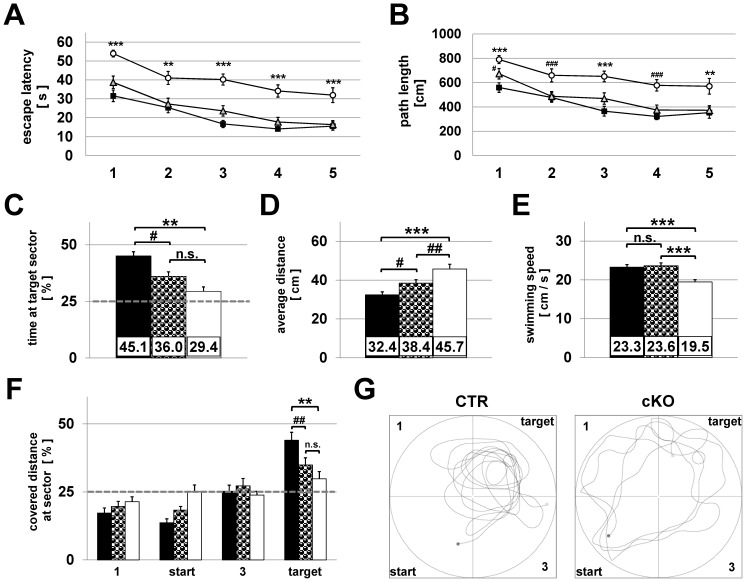
Morris Water Maze task. cKO mice show a significantly impaired space learning and spatial memory compared to HET and CTR animals. (A) & (B) During the first five days place-learning was analysed by assessing (A) the time and (B) the path-length to reach the hidden platform. Data of the acquisition trials were averaged across four trials per day. Data points are mean ± SEM (cKO: white circles, n = 18; HET: grey triangles, n = 19 and CTR: black squares, n = 20). (C) to (F) During Morris Water Maze retention trials, the platform was removed from the basin and spatial memory was analysed by assessing the mousés ability to recall the position of the formerly hidden platform. Means + SEM are shown. (C) Percentage of total time spent at the target sector for CTR (black, n = 20), HET (half tone, n = 19) and cKO mice (white, n = 18). (D) Average distance to platform position during retention trial. (E) Average swimming speed of mice during retention trial. (F) Percentage of distance covered at the four water maze sectors. Chance level is indicated by the dashed line. # *P*<0.05; ## *P*<0.03; ** *P*<0.003; *** *P*<0.001 (one-way ANOVA & Student-Newman-Keuls post hoc test). (G) Illustration of two representative swimming tracks of a CTR (left) and cKO animal (right) during the retention trial at day 6 of the Morris Water Maze experiment. Circles at the target sectors indicate the position of the platform during acquisition trials.

The Morris water maze is one of the most established spatial learning and memory tasks which is known to depend on hippocampal function [28]. During acquisition phase (d1-d5) cKO mice showed a significantly increased time to locate the platform (Fig. 7A). Furthermore, the average distances towards the platform were significantly longer for cKO mice compared to CTR and HET mice (Fig. 7B). During retention trial CTR mice spent 45.1±3.0% of total time in the target sector, whereas HET mice 36.0±3.0% and cKO mice only 29.4±2.8%, (Fig. 7C). The average distance of the animals to the platform position was 32.4±1.6 cm for CTR, 38.4±1.9 cm for HET and 45.7±2.5 cm for cKO mice (Fig. 7D). All groups differed statistically significantly from each other (*P*<0.05). CTR and HET animals swam with the same speed (23.3±0.7 vs. 23.6±0.8 cm/s), whereas swimming speed of cKO mice was significantly reduced to 19.5±0.6 cm/s (*P*<0,001) (Fig. 7E). To check whether the time spent at target sector was influenced by the animalś swimming-skills, we analysed in parallel the covered distance at each sector during the retention trial. The average covered distance at target sector by CTR (44.0±2.9%, *P*<0.003) and HET (34.9±2.6%, *P*<0.03) animals was significantly increased, whereas cKO mice operated at chance level (29.8±2.7%) (Fig. 7F). Figure 7G illustrates two representative swimming tracks. A Morris Water Maze cued learning task revealed no significant differences in cued learning performance as seen by analysing the parameters escape latency (Fig. S6A) and path length (Fig. S6B).

Of note, a few cKO mice occasionally showed seizures (3 out of 21 animals sporadically demonstrated seizures; data from these 3 animals were excluded from the analysis). We observed this phenotype only in the context with the Morris water maze, but not during other behavioural tests.

### Voluntary running wheel

**Figure 8:**
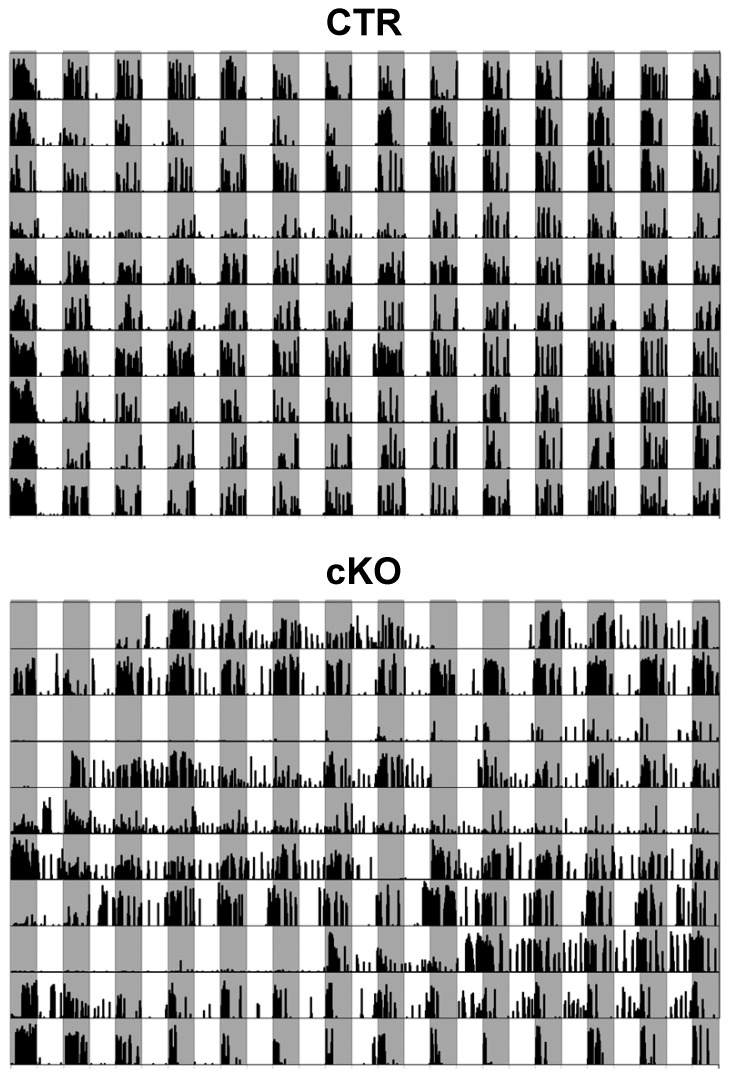
Circadian wheel running activity. Voluntary wheel running activity was recorded for 14 days for 10 CTR and 10 cKO mice (horizontal tracks). Each track shows running activity for 12 h nighttime (grey) and 12 h daytime (white). Within each area black bars indicate the percentage of running time shown for a 15-min time window each.

Since an increased spontaneous activity of cKO mice could influence the interpretation of above described tests, the animals were challenged with a voluntary running wheel experiment. During 14 days of the test all animals increased their running speed. CTR (N = 10) and HET (N = 11) mice improved their performance in the running wheel from 71.0±4.4 (HET 57.5±5.8) to 112.3±3.3 rpm (HET 106.0±4.2 rpm), whereas cKO (N = 10) mice increased their average speed from 55.5±8.0 to 89.1±5.1 rpm (Fig. S7). However, compared to CTR or HET mice, cKO animals did not show significant differences in the cumulative running times per day (Fig. S7), but demonstrated a noticeable variability for the use of the running wheel. Analysis of circadian wheel running behaviour revealed that cKO mice not only run at night but also 25.3±3.8% of their total-running time at daytime (Fig. 8 and Fig. S8). In contrast CTR and HET mice spent only 2.2±0.8% and 7.22±1.8% of total running time at daytime, respectively. These data indicate a strong attenuation of circadian rhythmicity in cKO animals.

This is a rather unexpected finding since in knock-out models Ca_V_2.1 has never been described to be involved in the maintenance of circadian rhythmicity *per se*, although a knock-in model in which a human familial hemiplegic migraine mutation was introduced showed an enhanced circadian phase setting phenotype [29].

## Discussion

Our electrophysiological investigation demonstrates that synaptic transmission at Schaffer collaterals to CA1 PC inputs in forebrain-specific conditional Ca_V_2.1 knock-out (cKO) mice is markedly diminished. We have tested this synapse because previous investigations have shown that in this region transmitter release depends on the expression of both, Ca_V_2.1 and Ca_V_2.2 channels [30]. Synaptic transmission evoked by single pulses in cKO mice was unaffected by the Ca_V_2.1 channel blocker ω-agatoxin IVA, but completely abolished by the Ca_V_2.2 blocker ω-conotoxin GVIA, confirming that Ca_V_2.1 channels at CA3-CA1 synapses are selectively impaired in this cKO model. Notably, multiple pulse stimulation at Schaffer collaterals of cKO mice induced a response which is more facilitating than in CTR mice. These results are consistent with previous investigations on selectively blockade of Ca_V_2 channels which demonstrated an enhanced paired-pulse facilitation mediated by Ca_V_2.2 channels [31]. This can be explained by presynaptic accumulation of Ca^2+^ resulting in an enhanced release probability.

The impaired transmission at glutamatergic synapses in the hippocampus led us to anticipate alterations of memory functions, changes in the activity as well as in anxiety-related behaviour. In fact, our cKO mice are characterized by a variety of memory deficits, such as a strong impairment of recognition and spatial memory, as evidenced by their particular performance in the object recognition and Morris water maze tasks. Object recognition has been shown a useful tool to assess behavioural and neuronal processes mediating storage and subsequent recall of features of known vs. new objects. It has also been considered a “non-matching-to-sample” learning task because evidence for object recognition involves more intense interaction with the new object compared to a familiar one [32]. In these tests, cKO mice were not able to differentiate between a familiar and a novel object. One might argue that an increased spontaneous drive or hyperactive-like behaviour of cKO mice influences the interpretation of our study. However, the results from the voluntary running wheel experiment and careful observation of our mice do not support this hypothesis.

During the Morris water maze experiment evaluation of the conventional parameter time indicates a rather strong impairment of place learning and spatial memory. The observed significantly reduced swimming speed of cKO mice may lead to a biased data interpretation. Therefore, we analysed additional parameters that are immune to swimming speed [33],[34]. All tested parameters – time and covered distance, as well as average distance to the platform position were highly correlative, indicating a strong impairment of spatial learning and memory of cKO mice. In addition the cued learning task further demonstrated intact abilities such as eye sight and adequate motor skills as well as normal motivation and an effective strategy to escape from the water [33].

During the open field and the elevated plus maze test cKO mice demonstrate an increased exploratory drive. The latter tests often have been used to investigate the anxiety level of mice and our data indicate that cKO mice show a decreased level of anxiety. However, we hypothesize that their behavioural phenotype is also caused by a strongly increased exploratory behaviour. An increased exploratory drive can be the result of an impaired working memory as also shown by hippocampal lesions in rodents [35], [36]. This interpretation is supported by the high number of crashes of cKO mice during the elevated plus maze experiment. The mice explore their environment driven by their native curiosity, but possibly do not correctly recall their environmental setup and typically crash when moving backwards. We assume that the impaired working memory of the mice which is also evident from the other behavioural tests, accounts for this behaviour. The increased exploratory drive in the open field arena and in the object recognition task may be explained in the same manner.

For a very limited number of cKO mice (below 10%) we occasionally observed a decreased threshold level of excitability for external stimuli, these animals were more sensitive to the experimental setup, demonstrated an escape behaviour and overreacted in response to stimuli such as touch or movement.

Intriguingly, the behavioural phenotype of our cKO mice shows many aspects which are comparable to the development of Alzheimeŕs disease (AD). Especially the initial symptoms of the disease are similar to the phenotypes of cKO mice [37]–[39]. The formation and accumulation of Aß oligomers represents a major contribution to the pathology of AD and it is assumed that extracellular and soluble Aß oligomers have numerous target sites at some distance from established plaques [40]. Ca_V_2.1 calcium channels may be involved in AD since oligomeric Aß suppresses Ca^2+^ current through Ca_V_2.1 channels either directly or indirectly via down-regulation of the channel [8], [41].

A major finding of our behavioural phenotyping of Ca_V_2.1 cKO mice is the result from the voluntary running wheel experiment, which represents a minimally invasive method evaluating the functionality of the circadian system [42]. Since cKO mice run not only during night but also at daytime, we conclude that they have an impaired circadian rhythmicity. None of the previously described global Ca_V_2.1 knock-out models pointed to this important role of Ca_V_2.1.

Ca^2+^ influx via voltage-gated calcium channels has been implicated in the coordination of gene expression and synchronization of rhythmicity in neurons of the suprachiasmatic nucleus (SCN), the master pacemaker of the brain. All major types of voltage-gated calcium channels have been found in the SCN [43]. L-type channels were the dominant family of voltage gated calcium channels, but only P/Q- and T-type channels, which were expressed at moderate levels, demonstrated rhythmic expression in the SCN. Although the specific functions of these calcium channels are not defined, it is believed that they are involved in regulating SCN neural activity and circadian behaviour. Circadian regulation of Ca_V_2.1 in SCN indicates an important role for the generation or coordination of oscillations in SCN clock cells and/or for the intercellular coupling between SCN neurons. The disruption of the circadian clock is one of the most common and earliest symptoms of AD [44]. Observed symptoms range from sleep disorders like insomnia or REM-sleep-behaviour disorders to the sundowning syndrome, a specific symptom often described in the context of dementia and particularly of AD [45], [46]. Again, these clinical observations are in line with the phenotype of Ca_V_2.1 cKO mice. The dramatically altered circadian rhythmicity of Ca_V_2.1 cKO mice represents a novel finding of our study since an impaired circadian system is thought to promote neurodegenerative processes in general [46].

In summary, our study gives novel insights into the manifold *in vivo* roles of the Ca_V_2.1 Ca^2+^ channel. Our conditional knock-out model enabled us to focus on the particular function of Ca_V_2.1 in the mouse forebrain for learning and memory. Future models based on local application of virus or vector-driven Cre expression will give a higher resolution on the physiological functions of Ca_V_2.1 in different brain regions.

## Supporting Information

Figure S1 **Western blot analysis.** (A) Forebrain-specific knock-out of Ca_V_2.1 was shown by hippocampus and neocortex preparations from male 132 day old CTR, HET and cKO mice. Preparations from cerebellum confirm an unaltered Ca_V_2.1 expression in cKO and HET mice. The Na^+^/K^+^-ATPase was used as loading control. Western blot was repeated three times (three mice per genotype). (B) Marginal compensatory up-regulation of Ca_V_2.2 and Ca_V_1.2 in the hippocampus in cKO compared to CTR mice. However, expression of Ca_V_2.2 and Ca_V_1.2 was not changed in neocortex. Western blot was repeated three times (three mice per genotype).
(TIF)

Figure S2 **Hanging wire test.** A hanging wire test did not show any difference in grip strength between adult male mice of all three genotypes (P = 0.282). Bar diagram shows the latency to fall off a wire lid (CTR: black n = 11, HET: half tone, n = 9, cKO: white, n = 9). Each mouse was tested in two trials (inter-trial interval 15 min, 60 second cut off time for the standard test-trial). Means + SEM are shown (Kruskal-Wallis one way ANOVA on ranks & Dunn's post hoc test).
(TIF)

Figure S3 **Body weight of CTR (black) HET (half tone) and cKO (white) mice.** Means + SEM are shown (A) cKO animals (n = 27) at an age of 50 days were marginally lighter than CTR (n = 27) and HET mice (n = 25). (B) Body weight of 110 day old mice did not differ (P = 0.141) between CTR (n = 15), HET (n = 12) and cKO animals (n = 11). ## *P*<0.03; ### *P*<0.01; *** *P*<0.001 (Kruskal-Wallis one way ANOVA on ranks & Dunn's post hoc test).
(TIF)

Figure S4 **Visual tracking drum test.** Visual performance of the animals was analysed by counting the head tracking movements (tracking movements/minute) in a rotating optical drum for two minutes (CTR black, n = 14; HET half tone, n = 9 and cKO white, n = 11). Means + SEM are shown (one-way ANOVA & Student-Newman-Keuls post hoc test; P = 0.815).
(TIF)

Figure S5 **Object recognition task.** Behaviour of mice (CTR (black, n = 18), HET (half tone, n = 15) and cKO mice (white, n = 18)) during the acquisition phase of the object recognition task. (A) Mice do not discriminate between two orbs during a 10 min acquisition trial in an object recognition task. The bar diagram shows the object interaction index (OI_index_), that represents the percentage of interaction time for one object and was calculated as follows: OI_index_ =  [T_orb1_/(T_orb1_ + T_orb2_)]×100 (T = object interaction time). Error bars indicate SEM. Chance level is indicated by the dashed line. (one-way ANOVA & Student-Newman-Keuls post hoc test; P = 0.562) (B) Cumulative object interaction time (means + SEM) with both objects during 10 min acquisition (Kruskal-Wallis one way ANOVA on ranks & Dunn's post hoc test P = 0.080).
(TIF)

Figure S6 **Morris Water Maze “cued learning task”.** The submerged platform was cued with a dark-grey object (cone) that extends above the water surface. Position of the platform and starting-position was changed during each trial to ensure that mice have to use the proximal cue to locate the submerged platform. Cued learning was analysed by assessing (A) the time and (B) the path-length to reach the platform at the fifth day of cued learning (CTR: black n = 8, HET: half tone, n = 4, cKO: white, n = 6). Means + SEM are shown (One-way ANOVA & Student-Newman-Keuls post hoc test, no significant differences, P values are shown on top of corresponding bar-diagram).
(TIF)

Figure S7 **Voluntary wheel running-activity.** Average running speed and time during a voluntary wheel running test. Data points are displayed as mean ± SEM. (A) The average running speed of CTR (black squares, n = 10), HET (white circles, n = 11) and cKO animals (white squares, n = 10) increased over 14 days of recording. (B) Average running time of CTR, HET and cKO mice during 14 days of voluntary wheel running were not significantly different (symbols and numbers of animals as in (A)). ## *P*<0.03; ### *P*<0.01; * *P*<0.005; ** *P*<0.003; *** *P*<0.001 (One-way ANOVA & Student-Newman-Keuls post hoc test).
(TIF)

Figure S8 **Voluntary wheel running of HET mice.** Voluntary wheel running activity of 11 HET mice recorded for 14 days (horizontal tracks). Each track shows running activity for 12 h nighttime (grey) and 12 h daytime (white). Within each area black bars indicate the percentage of running time shown for a 15-min time window each.
(TIF)
