# Correction: Ablation of Ca_V_2.1 Voltage-Gated Ca^2+^ Channels in Mouse Forebrain Generates Multiple Cognitive Impairments

**DOI:** 10.1371/annotation/d8de7f3c-6be2-44b6-b835-3cfd1518d7f5

**Published:** 2013-12-30

**Authors:** Robert Theodor Mallmann, Claudio Elgueta, Faten Sleman, Jan Castonguay, Thomas Wilmes, Arn van den Maagdenberg, Norbert Klugbauer

A funding organization for the second author, Claudio Eglueta, was incorrectly omitted from the Funding Statement. The Funding Statement should read: "This work was supported by a DFG grant to NK (SFB780, project A1) and the VW-Foundation to CE. The funders had no role in study design, data collection and analysis, decision to publish, or preparation of the manuscript."

In addition, affiliation number 2 for the second author is incorrect. The correct affiliation number 2 is: Institut für Physiologie I, Albert-Ludwigs-Universität, Freiburg, Germany.

In addition, all Supplementary Figure files are missing in both hyperlink and thumbnail format. The Supplementary Figures can be viewed at the following links, as indicated:

Figure S1: 

Figure S2: 

Figure S3: 

Figure S4: 

Figure S5: 

Figure S6: 

Figure S7: 

Figure S8: 
